# Dynamic Compensation Method for Humidity Sensors Based on Temperature and Humidity Decoupling

**DOI:** 10.3390/s22197229

**Published:** 2022-09-23

**Authors:** Wenxuan Yang, Wenchang Li, Huaxiang Lu, Jian Liu, Tianyi Zhang

**Affiliations:** 1 Key Laboratory of Solid-State Optoelectronics Information Technology, Institute of Semiconductors, Chinese Academy of Sciences, Beijing 100083, China; 2 University of Chinese Academy of Sciences, Beijing 100049, China; 3 Semiconductor Neural Network Intelligent Perception and Computing Technology Beijing Key Laboratory, Beijing 100083, China; 4 State Key Laboratory of Superlattices and Microstructures, Institute of Semiconductors, Chinese Academy of Sciences, Beijing 100083, China

**Keywords:** humidity sensor, fast response, dynamic compensation, temperature and humidity decoupling

## Abstract

Currently, integrated humidity sensors with fast-response time are widely needed. The most commonly used polyimide capacitive humidity sensor has a long response time, which is difficult to meet the need for a fast response. Most studies focusing on technology and materials have a high cost and are difficult to ensure compatability with the CMOS process. The dynamic compensation method can shorten the response time by only adding digital circuits or software processing. However, conventional compensation technology is not suitable for humidity sensors due to temperature coupling. This paper proposes a new dynamic compensation method for humidity sensors based on the decoupling of temperature factors by analyzing the coupling relationship between sensor dynamic characteristics and temperature. Simulations and experiments were used to verify the proposed method. The experimental results show that the proposed method reduces the humidity response time of the sensor by 85.6%. The proposed method can effectively shorten the response time of humidity sensors.

## 1. Introduction

The integrated sensors, which include sensing front-end circuits, an analog-to-digital converter, and a digital processing part, are developed rapidly due to their low cost, small size, and ease of use. Humidity sensors are widely used in many applications, such as environmental monitoring (e.g., indoor climate control in smart houses or industrial production), process control (e.g., high reliability in integrated circuit fabrication), medical equipment, and biotechnology [1,2,3,4,5]. In addition, sensors with a rapid response time are required in areas such as respiratory monitoring and semiconducting process control [6,7,8,9,10]. These scenarios require the response and recovery times of the humidity sensors to be around 1 s [10]. However, most commercial integrated humidity sensors have a response time of more than 8 s. Therefore, the above requirements could not be met.

There are two methods to achieve a fast-response humidity sensor. One way to improve the response speed is to increase the contact area between the probe and the air using specific process technologies, such as new moisture-sensitive materials, ultra-thin moisture-sensitive films, special moisture-sensitive probe structures, and surface treatment of the moisture-sensitive layer [6,7,8,9,10,11,12,13,14,15,16]. Uksong Kang et al. reported a capacitive humidity sensor that used multiple polyimide cylinders with a diameter of several microns as the probe [11]. This structure could help improve the sensor’s response time to 1 s. In the work of Nimmakayala V.V. Subbarao et al., an OFETs-based humidity sensor was fabricated [15]. The experiments showed that this material-based sensor demonstrated a response time of 0.73 s and a recovery time of 0.52 s. Cheng Zhou et al. proposed a new humidity sensor realized with nanowire arrays [8]. The nanowire arrays were fabricated on a flexible substrate. The sensor had a response time of 0.63 s and was used in respiratory monitoring. A polyelectrolyte-based humidity sensor with a response/recovery time of 0.29/0.47 s is presented by Jianxun Dai et al. [10]. Although special process technologies can improve the response time of humidity sensors, such a method can increase the process complexity and cost, making it difficult to be compatible with the CMOS process. Therefore, it is difficult to be used in integrated humidity sensors.

The other way to improve the response time is to compensate the sensor output dynamically. Dynamic distortions are compensated by digitally processing the sensor output, and results can be obtained as close as possible to the actual measurement values [17,18,19,20]. This method only requires adding part of the digital circuits, which is easy to implement in the CMOS process. Dynamic compensation technology has been employed in temperature sensors [21] and stress and pressure sensors [22,23,24]. The models of these sensors can be regarded as a linear time-invariant (LTI) system. However, humidity sensors are not linearly time-invariant with respect to temperature changes due to temperature coupling. Therefore, applying the existing dynamic compensation methods to humidity sensors is challenging.

This paper aims to solve the effect of temperature coupling on humidity sensors and improve the response time with a dynamic compensation method. A dynamic compensation algorithm is proposed based on temperature and humidity decoupling by modeling and analyzing the coupling effect of temperature on sensing humidity. After decoupling, the sensor system is transformed into a linearly time-invariant system, and the dynamic compensation is realized. The rest of this study is organized as follows. First, Section 2 analyzes the temperature coupling effect in the humidity-sensing process. Second, the overall principles of the proposed compensation are explained in Section 3. Then, simulation and experimental results are provided in Section 4. Section 5 discusses humidity sensor recovery time and the long-term stability of dynamic characteristics. Finally, the conclusion is presented in Section 6.

## 2. Coupling Effect of Temperature on Humidity Sensing

### 2.1. Humidity-Sensing Mechanism

**Figure 1:**
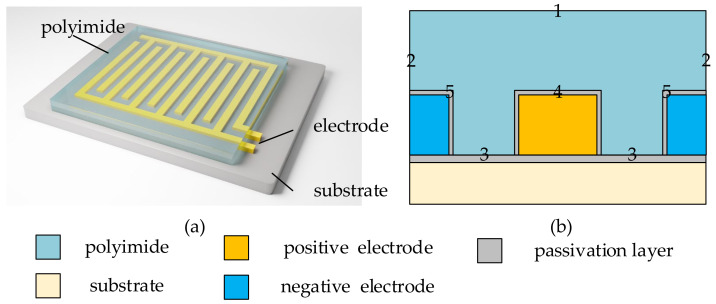
The medium and probe structure of the humidity sensor used in this paper. (**a**) The structure of the polyimide probe with comb-shaped electrodes. (**b**) The schematic diagram of the longitudinal section of the adjacent electrodes in the probe. Boundary 1 is the contact surface between the probe and the air. Boundary 2 is the symmetrical contact surface between the probe and the adjacent medium. Boundary 3 is the contact surface between the probe and the substrate passivation layer, Boundary 4 is the positive electrode passivation layer, and Boundary 5 is the negative electrode passivation layer.

First, the humidity-sensing process is analyzed. The media and structures of capacitive humidity sensors are various. This paper studies a common type of sensor as an example. As shown in Figure 1, the medium of this sensor is polyimide, and its probe consists of comb-shaped electrodes. The concentration of water molecules in the air above the electrodes varies with the relative humidity or temperature of the environment. When the concentration of water molecules changes, the dynamic diffusion equilibrium between water molecules in the pores of the polyimide medium and those in the air is disrupted. As a result, water molecules begin to diffuse in one direction. The diffusion direction and speed are determined by the temperature and the difference in the concentration of water molecules in the medium and the air. Therefore, the concentration of water molecules in the medium changes. Furthermore, since water molecules distributed in the polyimide medium are electrically polarized by an external electric field, the concentration of water molecules determines the capacitance value of the probe.

In order to catch the main factors affecting the dynamic characteristics of a sensor, the following assumptions are made:

i. Medium is homogeneous. A polyimide probe is a uniformly isotropic porous medium with the same porosity. The change in diffusivity is consistent with temperature. Gases, including water vapor in the medium, are ideal gases that do not react with the polyimide medium. Ignoring the diffusion resistance of the film surface, the water vapor concentration at the contact position between the medium and the air is uniform and the same as those in the ambient air.
ii. The temperature is uniform. In polyimide probes, the diffusion equations for temperature transfer and water molecule transfer take the same form. However, the thermal diffusivity in polyimide and silicon wafers is much greater than the water molecule diffusivity in polyimide. As shown in Table 1, some materials have thermal diffusivities that are 106 times higher than the diffusivity of water molecules. Assuming a response time of 10 s for the humidity sensor, the thermal diffusion time is less than 1 ms. Therefore, when modeling the dynamic process of water molecule diffusion, the temperature in the sensor Tsensors can be considered to be uniform.
iii. Signal processing time can be ignored. This is because the time required for signal amplification and digitization of the probe is milliseconds.

**Figure 2:**
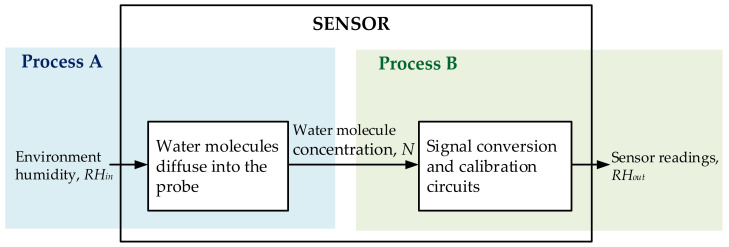
Decomposition of humidity-sensing process.

As shown in Figure 2, the humidity-sensing process is divided into two parts. In Process A, the temperature and humidity changes in the air determine the concentration of water molecules in the probe. The dynamic change process of environmental temperature and humidity affects the distribution of water molecules in the probe. In Process B, the current concentration of water molecules in the probe determines the sensor reading. Considering that the signal processing and conversion times are negligible, this process can be considered static.

The integrated humidity sensor is calibrated so that the sensor readings correspond to the correct water molecule concentration in the probe. Therefore, the concentration of water molecules in the probe corresponds linearly to the sensor readings in Process B. Process A, in which atmospheric water molecules diffuse into the probe medium, is the main process affecting the dynamic properties of the humidity sensor.

### 2.2. Coupling Effect of Temperature on Sensing Humidity

#### 2.2.1. Process A

**Figure 3:**
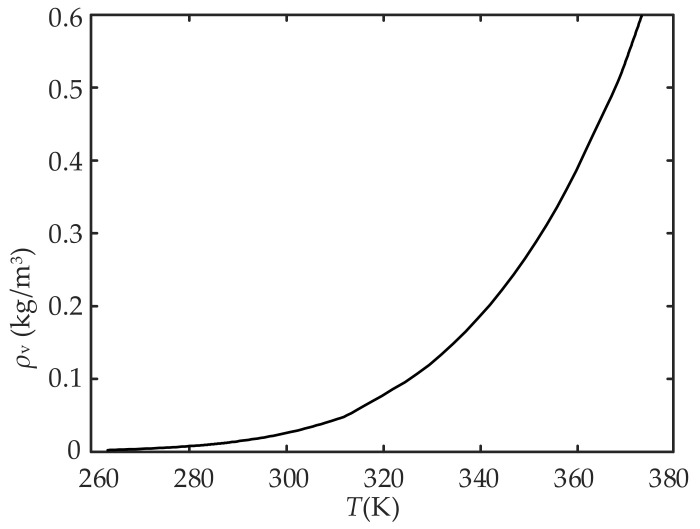
The relationship between *ρ*_v_(*T*) and *T*.

The relationship between the concentration of water molecules in the air and the humidity is given by Equation (1):

(1) $$RH_{in}=N_{in}M/\rho_{v}(T)$$

where *N*_in_ is the concentration of water molecules in the air, *RH*_in_ is the percentage of ambient relative humidity, *ρ*_v_(*T*) is the density of saturated vapor at temperature *T*, which varies with *T*, and *M* is the molar mass of water. The curve of *ρ*_v_(*T*), as a function of temperature, is shown in Figure 3. Figure 3 and Equation (1) show that *RH*_in_ changes with *ρ*_v_(*T*) even if *N*_in_ is constant, i.e., *RH*_in_ itself is coupled with temperature.

The change in *N*_in_ diffuses into the polyimide layer of the probe. This diffusion of water molecules can be described according to Fick’s law as follows:

(2) $$\frac{\partial N}{\partial t}=D_{e}(\frac{\partial^{2}N}{\partial x^{2}}+\frac{\partial^{2}N}{\partial y^{2}}+\frac{\partial^{2}N}{\partial z^{2}})$$

where *t* is the diffusion time, *N* is the concentration of water molecules in the polyimide probe, *N* = *N*(*x*, *y*, *z*, *t*_present_), and *D*_e_ is the effective diffusion coefficient of water molecules diffusing through the polyimide. The boundaries of the polyimide medium can be divided into two parts. One boundary (*S_air_*) is in contact with the air (Boundary 1 in Figure 1). The other (*S_close_*) is the boundary where no exchange of water molecules occurs (Boundaries 2 to 5 in Figure 1, including Boundary 2 for symmetry). Ignoring the force exerted on the water molecules that pass through *S_air_* and enter the air-medium film, the boundary conditions for Equation (2) can be written as follows:

(3) $${\left.N\right|}_{(x,y,z)\in S_{air}}=N_{in}(t)$$

(4) $${\left.\frac{\partial N}{\partial x}\right|}_{(x,y,z)\in S_{close}}={\left.\frac{\partial N}{\partial y}\right|}_{(x,y,z)\in S_{close}}={\left.\frac{\partial N}{\partial z}\right|}_{(x,y,z)\in S_{close}}=0$$

(5) $${\left.N\right|}_{t=0}=N(x,y,z,0)$$

where *N*(*x*, *y*, *z*, 0) is the concentration of water molecules in the polyimide probe when *t* = 0. Process A is a definite solution problem consisting of the diffusion Equation (2) and boundary conditions Equations (3) to (5). The coupling effect of temperature on humidity sensing is found to be mainly in two aspects:

i. According to Equation (1), *RH*_in_ is affected by both temperature and *N*_in_.
ii. Since *D*_e_ is affected by temperature, the diffusion Equation (2) is coupled with temperature. The relationship between *D*_e_ and temperature is written as follows [25]:

(6) $$D_{e}=D_{e0}{\left(T/T_{0}\right)}^{1.75}$$

where *T*_0_ is the reference temperature, and *D*_e0_ is the diffusion coefficient at the reference temperature.

The above two coupling influences are present in most humidity sensors. The first influence is suitable for humidity sensors measuring relative humidity. The second influence is suitable for most humidity sensors because even though different humidity sensors convert humidity into different physical quantities, most sensors measure humidity depending on the concentration of water molecules in the moisture-sensitive material. For water molecules diffusing through different moisture-sensing materials, only the relationship between diffusion coefficient and temperature differs from Equation (6).

#### 2.2.2. Process B

Process A determines the water molecule concentration distribution in the PI membrane, since water molecules are polarized in an electric field, a change in water molecule concentration *N* changes the probe’s dielectric constant, leading to a change in the probe’s capacitance. The capacitance of the probe can be obtained from the empirical formula for the relationship between dielectric constant change, water molecule concentration, and the electrostatic field equation [26]. The above empirical formula can be obtained by substituting Equations (8)–(10) into Equation (7).

(7) $$\varepsilon_{PI}^{*}={\left[\gamma (\varepsilon_{H_{2}O}^{1/3}-\varepsilon_{PI}^{1/3})+\varepsilon_{PI}^{1/3}\right]}^{3}$$

(8) $$\varepsilon_{PI}=2.93$$

(9) $$\varepsilon_{H_{2}O}=78.54\{1-4.6\times 10^{-4}(T-298)+8.8\times 10^{-6}{\left(T-298\right)}^{2}\}$$

(10) $$\gamma =0.0404\times [1-0.00243\times (T-298)]{\mathrm{RH}}^{0.836\times \frac{1-2.22\times 10^{4}(T-298)+2.34\times 10^{5}{\left(T-298\right)}^{2}}{1+0.0049\times \exp[0.12\times (T-298)]}}$$

where ε_PI_^*^ is the relative permittivity for the mixture of polyimide and water, *ε_PI_* is the relative permittivity for pure polyimide, and *ε_H__2__O_* is the relative permittivity for water.

The concentration distribution of water molecules in the PI film determines the dielectric constant distribution of the capacitive medium of the probe. After determining the boundary conditions of the electric field (potential difference between the positive and negative electrodes, grounding of the substrate, etc.), the electric displacement vectors ***D*** and the electric field strength ***E*** can be obtained through the electrostatic field equation:

(11) $$\nabla \cdot D=\rho_{f}$$

(12) $$E=\frac{D}{\varepsilon_{PI}^{*}\varepsilon_{0}}$$

where *ρ*_f_ is the surface density of free charge, which is 0 inside the PI film. From the above results, we can integrate the entire domain Ω to find the electrostatic energy *W* carried by the capacitor:

(13) $$W=\int_{\Omega }D\cdot Ed\Omega$$

So far, we can find the capacitance value:

(14) $$C=\frac{2W}{U^{2}}$$

where *U* is the potential difference between the electrodes.

**Figure 4:**
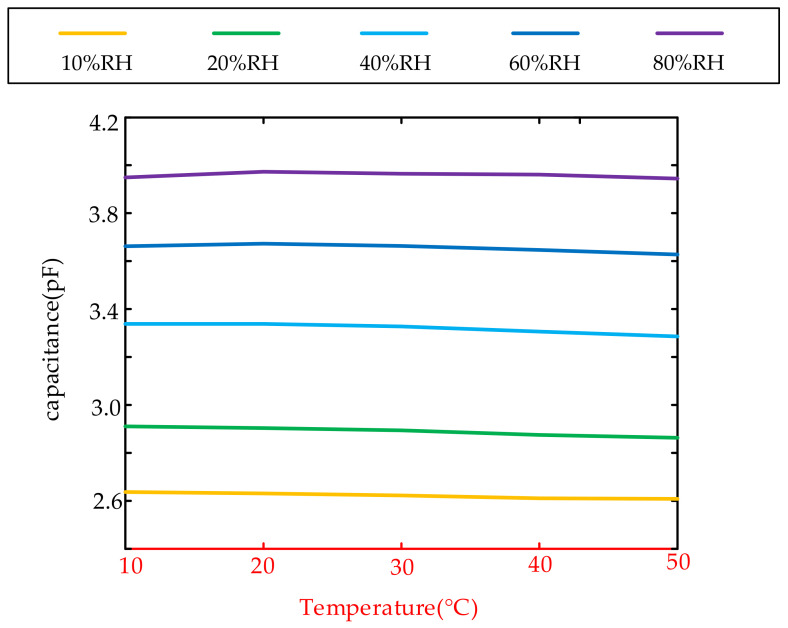
Relationship between Polyimide Humidity Capacitor and Temperature and Humidity.

We tested the capacitance value of the capacitance probe samples we designed and tape-out under different temperatures and humidity. Figure 4 is the average result of 7 samples.

It can be seen from Figure 4 that the capacitance value of our capacitance probe sample is hardly affected by temperature, and the main influencing factor is the relative humidity value. Under the condition that the geometry of the probe does not change, it shows that the dielectric constant of the polyimide film coated on the probe also mainly changes with relative humidity, but hardly changes with temperature.

The temperature and humidity characteristic curve must be calibrated before the temperature and humidity sensor leaves the factory. The probe capacitance hardly changes with the temperature, which greatly reduces the calibration cost of the humidity sensor. The calibrated temperature and humidity sensor ensures the accuracy of the output results in the steady state and also calibrates the measurement deviation caused by the nonlinearity of the PI dielectric constant or the influence of temperature in Process B. Therefore, the dynamic characteristics of a well-calibrated humidity sensor are negligibly affected by the factors in Process B.

## 3. Decoupling-Based Dynamic Compensation Method

### 3.1. The Principle of Dynamic Compensation for LTI System

**Figure 5:**
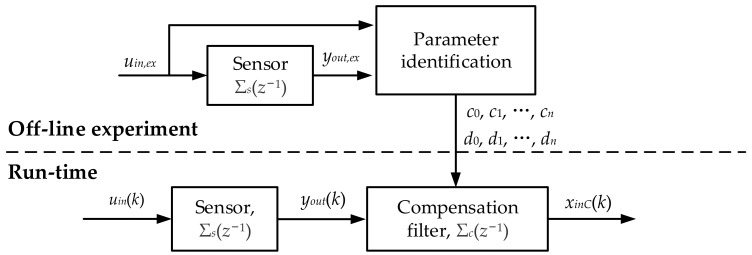
LTI system dynamic compensation process.

The conventional dynamic compensation method for an LTI system is introduced in this section firstly. The compensation process is shown in Figure 5. The transfer function of a sensor and compensation filter are Σ_s_(*z*^−1^) and Σ_c_(*z*^−1^), respectively. The dynamic characteristics of the sensor Σ_s_(*z*^−1^) can be compensated using a series compensation filter Σ_c_(*z*^−1^). The parameters of Σ_c_(*z*^−1^) can be obtained by parameter identification from the experimental data *u_in,ex_* and *y_out,ex_* during the off-line stage. The specific solution method is explained in the next section.

Suppose that the compensation filter transfer function is as follows:

(15) $$\Sigma_{C}(z^{-1})=\frac{c_{0}+c_{1}z^{-1}+\cdots +c_{n}z^{-n}}{1+d_{1}z^{-1}+\cdots +d_{n}z^{-n}}$$

The output compensation series is as follows:

(16) $$x_{inC}(k)=c_{0}y_{out}(k)+c_{1}y_{out}(k-1)+\cdots +c_{n}y_{out}(k-n)-d_{1}x_{inC}(k-1)+\cdots +d_{n}x_{inC}(k-n)$$

The case of *k* − *n* ≤ 0 occurs when compensating for the first few samples, so we need the given pre-measurement data. In this case, the sensor is in a steady state before the measurement, i.e., when *k* − *n* ≤ 0.

(17) $$x_{inC}(k-n)=y_{out}(k-n)=y_{out}(1)$$

### 3.2. Solving the Compensation System

The purpose of solving the compensation system is to obtain the parameter of the compensation filter. The most direct way to solve the compensation filter is to find the inverse system of the sensor system Σ_s_(*z*^−1^). However, there are cases where the inverse system Σ_s_^−1^ (*z*^−1^) does not exist or causes divergence in compensation results. In this case, an optimal approximation of the inverse system Σ_s_^−1^ (*z*^−1^) can be obtained by the least-squares method or particle swarm optimization to ensure convergence of the compensation system.

In order to identify the parameters of Σ_c_(*z*^−1^), an offline experiment is performed. In the experiment, a controlled input series *u_in__,ex_*(*k*) and the output series *y_out,ex_*(*k*) of the sensor are obtained as the apriori information for the parameter identification. The aim of the parameter identification is to make the compensated series *x_in_*_C_(*k*) most approximate to the input series *u_in__,ex_*(*k*).

Considering the feasibility of the experimental design and the accuracy of the compensation, the step-change condition of the external environmental humidity is adopted in our experiment. To identify the parameters of Σ_c_(*z*^−1^), the order of Σ_c_(*z*^−1^), or *n* in (15), must first be determined. After comparing the residuals of models of different orders, the appropriate model order can be judged. Once the model order is determined, the difference equation for the transfer characteristics of the digital compensation filter can be expressed as follows:

(18) $$y_{out}(k)+d_{1}y_{out}(k-1)+\cdots +d_{n}(k-n)=c_{0}x_{inC}(k)+c_{1}x_{inC}(k-1)+\cdots +c_{n}x_{inC}(k-n)$$

Our goal is to find the optimal solution for the digital compensation filter parameters to minimize the following objective function *J*

(19) $$J=\sum_{N}^{k=1}{\left(\Sigma_{c}^{d}(z^{-1})y_{out}(k)-\Sigma_{c}^{c}(z^{-1})x_{in}(k)\right)}^{2}$$

where, Σ_c_^d^(*z*^−1^) = *1* + *d*_1_*z*^−1^ + … + *d_n_z*^−n^, and Σ_c_^c^(*z*^−1^) = *c_0_* + *c_1_z*^−1^ + … + *c_n_z*^−n^. In this paper, particle swarm optimization is used to solve the optimal approximation of Σ_c_(*z*^−1^).

It should be noted that due to the temperature influence on the humidity sensor output, the solution of Σ_c_^d^(*z*^−1^) and Σ_c_^c^(*z*^−1^) for a humidity sensor only corresponds to a single temperature. Namely, every temperature needs a new series of compensation parameters. Therefore, the conventional method is not suitable for humidity sensors.

### 3.3. Dynamic Compensation Based on Temperature and Humidity Decoupling

**Figure 6:**
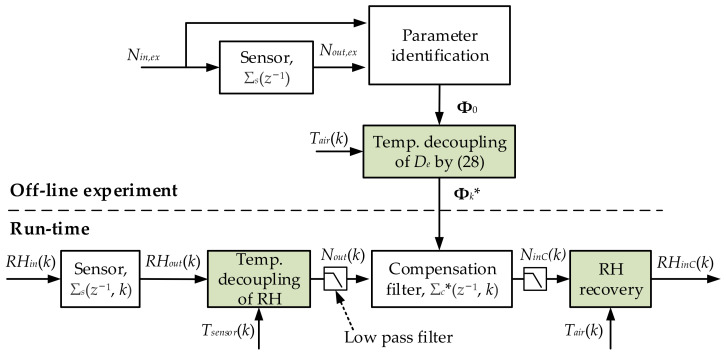
Dynamic compensation process for humidity sensors based on temperature decoupling.

A new dynamic compensation method based on temperature and humidity decoupling is proposed in this section. To eliminate the influence of temperature coupling factors, two decoupling procedures are designed to ensure an effective compensation when the temperature changes. Figure 6 shows the overall compensation process for the humidity sensor. 

The temperature decoupling of RH is performed before the compensation filter. The parameters of the compensation filter are also obtained from an offline experiment. During the experiment, temperature decoupling of *D*_e_ is introduced. These two decoupling procedures will be introduced in two subsections of this section. The low-pass filters are added to the compensation process to reduce the high-frequency noise amplified by dynamic compensation.

#### 3.3.1. Temperature Decoupling of RH

As described in Section 3, relative humidity is related to temperature while water molecule concentration is not, so the temperature factor in RH can be decoupled by converting the compensation variable from relative humidity to water molecule concentration. The relative humidity output of the humidity sensor *RH_out_*(*k*) can be converted into the water molecule concentration output *N_out_*(*k*) by Equation (1). After the compensation is completed, the compensated results *N_in_*_C_(*k*) are restored to the relative humidity result *RH_in_*_C_(*k*). 

It should be emphasized that the temperature used during RH decoupling is the temperature measurement results of the sensor, *T*_s*en*s*or*_, and the temperature used during RH recovery is the ambient air temperature *T_air_*. Because *T*_s*en*s*or*_ is usually delayed from actual ambient air temperature, *T_air_* is obtained by dynamic compensation of *T*_s*en*s*or*_ using the conventional method described in Section 3.1 and Section 3.2.

#### 3.3.2. Temperature Decoupling of D_e_

After the temperature decoupling of RH, it is only necessary to focus on the diffusion coefficient *D_e_*. It can be seen that temperature affects the diffusing process of water molecules through *D*_e_ in Equation (2). The method of decoupling the temperature factor of *D*_e_ is to use variable substitution so that an equivalent diffusing process is not affected by temperature.

Variable substitution:

(20) $$t^{*}=\int_{0}^{t}{\left(T(\tau )/T_{0}\right)}^{1.75}d\tau$$

where *T*_0_ is the reference temperature. Then, the following relationship is obtained

(21) $$\frac{\partial N}{\partial t}=\frac{\partial N}{\partial t^{*}}\frac{dt^{*}}{dt}=\frac{\partial N}{\partial t^{*}}{\left(T(t)/T_{0}\right)}^{1.75}=\frac{\partial N}{\partial t^{*}}\frac{D_{e}}{D_{e0}}$$

Substitute (21) into the diffusion Equation (2) to obtain

(22) $$\frac{\partial N}{\partial t^{*}}=D_{e0}(\frac{\partial^{2}N}{\partial x^{2}}+\frac{\partial^{2}N}{\partial y^{2}}+\frac{\partial^{2}N}{\partial z^{2}})$$

After replacing time *t* with t*, Equation (22) is not affected by temperature, i.e., the sensor system with time t* (regarded as Σ_s_^*^(s)) has the same parameters as the system with time *t* at the temperature *T*_0_ (regarded as Σ_s0_(s)). The meaning of the variable substitution is to make the time change rate of t* proportional to *D_e_* so that the diffusion Equation (22) with time t* is not affected by temperature. In the following discussion, t* refers to the time after substitution.

Because the diffusing process of a humidity sensor in the t* domain is temperature independent, the sensor system Σ_s_^*^(s) can be compensated by a method similar to the conventional technology. However, since the sampling of Σ_s_^*^(s) in the t* domain is unequally spaced, the parameters of compensation filter Σ_c_^*^ are not constant. The compensation filter system is regarded as Σ_c_^*^(*z*^−1^, *k*). As shown in Figure 6, the temperature decoupling of *D*_e_ works during the offline experiment to obtain the variable compensation parameters. 

The relationship between the sensor systems Σ_s_^*^(s) and Σ_s0_(s) discussed earlier is for a continuous system. When time *t* is replaced by t*, the temperature factor is decoupled, but the compensation filter system Σ_c_^*^(*z*^−1^, *k*) must be solved at t* to compensate the sensor. It is important to note that when t* replaces *t*, the sampling points correspond one-to-one, and the sampled sensor systems Σ_s_^*^(*z*^−1^, *k*) and Σ_s_(*z*^−1^, *k*) are identical. Therefore, Σ_c_^*^(*z*^−1^, *k*) can be used to compensate the sensor system Σ_s_(*z*^−1^, *k*) with time *t*.

**Figure 7:**
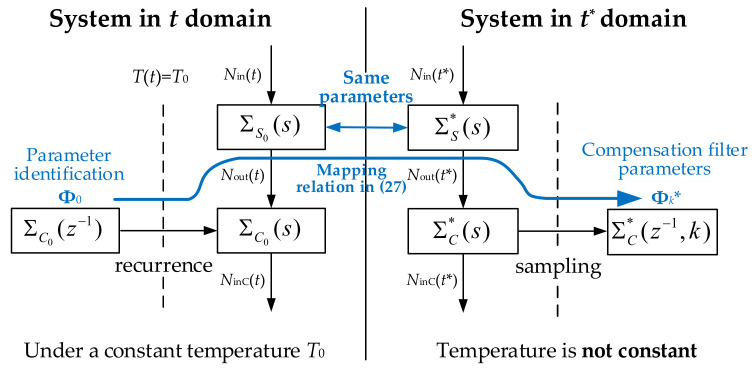
The relationship between several systems after substitution and sampling.

The required compensation system Σ_c_^*^(*z*^−1^, *k*) can be obtained by the compensation system Σ_c0_(*z*^−1^) at a constant temperature. Σ_c0_(*z*^−1^) can be solved by the conventional method. The process of solving Σ_c_^*^(*z*^−1^, *k*) from Σ_c0_(*z*^−1^) is shown in Figure 7. Σ_s0_(s) and Σ_c0_(s) are the continuous sensor and compensation systems at time *t* and temperature *T*_0_. Σ_s_^*^(s) and Σ_c_^*^(s) are the continuous sensor and compensation systems at time t^*^. It should be noted that Σ_s0_(s) and Σ_s_^*^(s) have the same mathematical form. Σ_c_^*^(*z*^−1^, *k*) can be sampled by Σ_c_^*^(s), and Σ_c0_(s) can be recurrenced from Σ_c0_(*z*^−1^). *k* represents the change in the transfer function of the system over time. *N_in_*(*t*), *N_out_*(*t*), and *N_in_*_C_(*t*) are the environmental input, sensor output, and compensation signals of water molecule concentration, respectively. 

Now we specifically solve Σ_c_^*^(*z*^−1^, *k*) from Σ_c0_(*z*^−1^). Suppose the identified Σ_c0_(*z*^−1^) as follows:

(23) $$\Sigma_{C_{0}}(z^{-1})=\frac{c_{0}+c_{1}z^{-1}+\cdots +c_{n}z^{-n}}{1+d_{1}z^{-1}+\cdots +d_{n}z^{-n}}$$

Information is lost during the sampling, and the state of the system at each moment cannot be known. To solve Σ_c_^*^(*z*^−1^, *k*), the temperature *T*(t*) at each moment must be given continuously. Therefore, the temperature at each moment must be estimated. For simplicity, we assume that the temperature between two samples is equal to the temperature measured in the previous sample, which is expressed as follows:

(24) T(t)=T(k),t∈(kΔt−Δt,kΔt)

Considering *T*(1) as time *t* = 0, the sampled t*(*k*) can be obtained from Equation (20) as follows:

(25) $$t^{*}(k+1)=\sum_{i=1}^{k}{\left(T(i)/T_{0}\right)}^{1.75}\Delta t$$

Substitute the relationship between t* and *t* into *T*(*t*) to obtain *T*(t*).

The sampling time is related to:

(26) $$\Delta t^{*}(k)=t^{*}(k+1)-t^{*}(k)={\left(T(k)/T_{0}\right)}^{1.75}\Delta t$$

We need to solve the following transfer function of the non-uniform sampling compensation system.

(27) $$\Sigma_{C}^{*}(z^{-1},k)=\frac{c_{0}(k)+c_{1}(k)z^{-1}+\cdots +c_{n}(k)z^{-n}}{1+d_{1}(k)z^{-1}+\cdots +d_{n}(k)z^{-n}}$$

In the subsequent analysis, the state-space model of the system is used. The state-space models corresponding to the transfer functions Σ_c0_(*z*^−1^) and Σ_c_^*^(*z*^−1^, *k*) are as follows:

(28) $$\Sigma_{C_{0}}(z^{-1}):\{\begin{array}{c} x(k+1)=Ax(k)+BN_{out}(k)\\ N_{inC}(k)=Cx(k)+DN_{out}(k) \end{array}$$

(29) $$\Sigma_{C}^{*}(z^{-1},k):\{\begin{array}{c} x(k+1)=A_{k}x(k)+B_{k}N_{out}(k)\\ N_{inC}(k)=Cx(k)+DN_{out}(k) \end{array}$$

where ***x***(*k*) is the state variable. ***A***, ***B***, C, and ***D*** are the state-space model parameters obtained from the transfer function parameter in Equation (23). We aim to solve the parameters ***A****_n_*, ***B****_n_*, C, and ***D*** in Σ_c_^*^(*z*^−1^, *k*).

The temperature and humidity output by the sensor between the two sampling points is assumed to be constant with the results of the previous sampling point. The compensation system is an LTI system with constant inputs between the two sampling points. The state transition matrix (STM) can represent the system state change between the two sampling points. The STMs at the *t* domain and t* domain are as follows.

(30) $$\left[\begin{array}{l} x(t(k)+\tau )\\ N_{out}(k) \end{array}\right]=\Phi^{\tau }\left[\begin{array}{l} x(t(k))\\ N_{out}(k) \end{array}\right]$$

(31) $$\left[\begin{array}{l} x(t^{*}(k)+\tau^{*})\\ N_{out}(k) \end{array}\right]=\Phi^{\tau^{*}}\left[\begin{array}{l} x(t^{*}(k))\\ N_{out}(k) \end{array}\right]$$

Since Σ_s0_(s) and Σ_s_^*^(s) are identical in physics, the corresponding Σ_c0_(s) and Σ_c_^*^(s) have the same parameters, and **Φ**^τ^ is equal to **Φ**^τ*^.

Substitute *τ* = Δ*t* and τ^*^ = Δt^*^(*k*) into Equations (30) and (31), respectively. The following relationships are obtained.

(32) $$\left[\begin{array}{l} x(k+1)\\ N_{out}(k) \end{array}\right]=\Phi^{\Delta t}\left[\begin{array}{l} x(k)\\ N_{out}(k) \end{array}\right]=\Phi_{0}\left[\begin{array}{l} x(k)\\ N_{out}(k) \end{array}\right]$$

(33) $$\left[\begin{array}{l} x(k+1)\\ N_{out}(k) \end{array}\right]=\Phi^{\Delta t^{*}(k)}\left[\begin{array}{l} x(k)\\ N_{out}(k) \end{array}\right]=\Phi_{k}^{*}\left[\begin{array}{l} x(k)\\ N_{out}(k) \end{array}\right]$$

**Φ**_0_ can be obtained from the parameters of the state-space model in Equation (28).

(34) $$\Phi_{0}=\left[\begin{array}{cc} A & B\\ 0 & 1 \end{array}\right]$$

The relationship between **Φ**_k_^*^ and **Φ**_0_ is as follows:

(35) $$\Phi_{k}^{*}=\Phi^{\Delta t^{*}(k)}=\Phi^{\Delta t(\Delta t^{*}(k)/\Delta t)}=\Phi_{0}{}^{\Delta t^{*}(k)/\Delta t}=\Phi_{0}{}^{{\left(T(k)/T_{0}\right)}^{1.75}}$$

Then

(36) $$\left[\begin{array}{cc} A_{k} & B_{k}\\ 0 & 1 \end{array}\right]=\Phi_{k}^{*}=\Phi_{0}{}^{{\left(T(k)/T_{0}\right)}^{1.75}}$$

The parameters of the system Σ_c_^*^(*z*^−1^, *k*) in Equation (27) can be obtained from ***A****_k_*, ***B****_k_*, ***C***, and ***D***.

## 4. Simulations and Experiments

### 4.1. Simulations

The finite element method is used to simulate the operating process of the humidity sensor, and the software used for the simulation is COMSOL Multiphysics. The dynamic output results of the humidity sensor under different temperatures and humidity are obtained and compensated by the dynamic compensation methods. Finally, the compensation results are compared with the given environmental humidity changes. To verify the effectiveness of the proposed dynamic compensation method, it is compared with the conventional method [23].

The simulation uses a polyimide humidity sensor in Figure 1 as an example. The simulation of the humidity-sensing process for this sensor is divided into three parts. The first is to simulate the diffusion process of water molecules in the probe under the corresponding temperature and humidity environment. The second is to simulate the electric field in the probe and obtain the capacitance change of the probe. The third is to simulate the built-in digital calibration of the humidity sensor through the difference between humidity, capacitance, and temperature under steady-state conditions.

**Figure 8:**
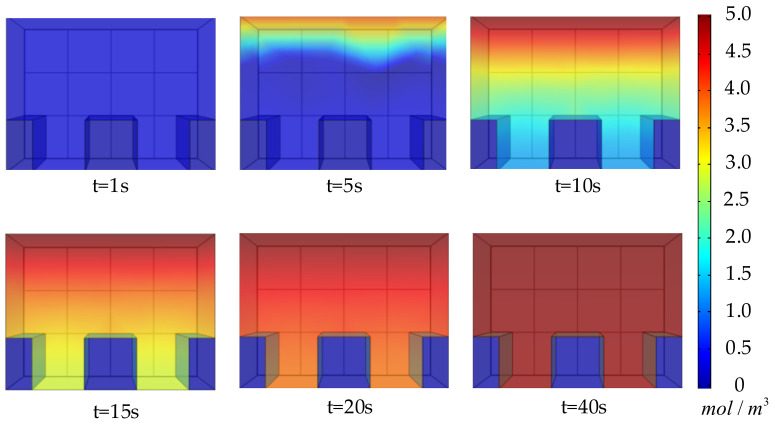
Simulation of the process of changing *N* with step changes in ambient humidity.

The simulation equations and boundary conditions for the diffusion of water molecules in the probe use the definite solutions given by Equations (2) to (5). For simplicity, the sensor temperature is considered to be the same as the ambient temperature in the simulation. Figure 8 shows that during the simulation, the specific change process of the water molecule concentration distribution *N* in the probe changes over time as the air humidity changes (increases) stepwise.

After the water molecule concentration distribution is obtained, the dielectric constant distribution in the probe can be obtained from Equations (7) to (10), and the capacitance value of the probe can be obtained through Equations (11) to (14). In the actual sensor, the humidity output is obtained through signal processing and digital calibration of the capacitance change of the probe. In the simulation, we realize the function by mapping the capacitance and temperature to the humidity. This function is obtained by interpolating the capacitance value of each temperature and humidity point in a steady state. 

**Figure 9:**
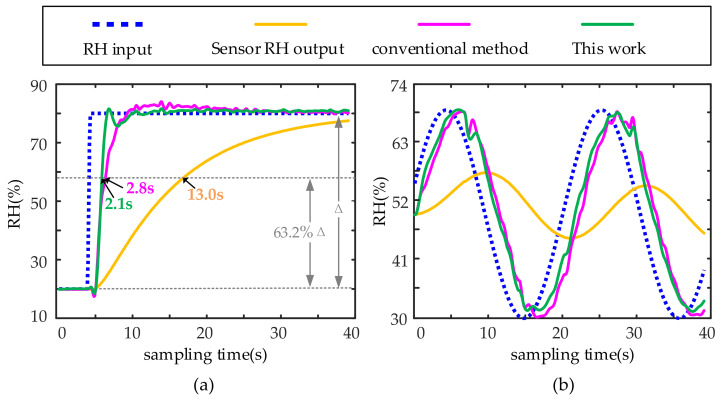
Simulation compensation results at a constant temperature as the reference temperature: (**a**) step change in ambient humidity and (**b**) periodic change in ambient humidity. The sampling frequency in the simulation is 10 Hz.

The first verification is carried out when the temperature is constant, and the humidity changes. Two humidity change forms are considered. One is a step change, and the other is a sinusoidal change. The results are shown in Figure 9. Parameters of the compensation filter Σ_c0_(*z*^−1^) are as follows:

(37) $$\Sigma_{C_{0}}(z^{-1})=\frac{52.5051-100z^{-1}+47.5374z^{-2}}{1-1.77854z^{-1}+0.82069z^{-2}}$$

Figure 9 shows that when the temperature is constant, both the conventional method and the proposed method can compensate for the output of the humidity sensor to make it close to the changes in ambient humidity. The response time is *t*_63.2%_, that is, the time when the output response reaches 63.2% of the input response Δ. The response time of the sensor system in Figure 9a is 13 s, and the response time after compensation by the conventional method is 2.8 s. The response time after compensation of the proposed method is 2.1 s, which shortens the sensor response time by 83.8%.

**Figure 10:**
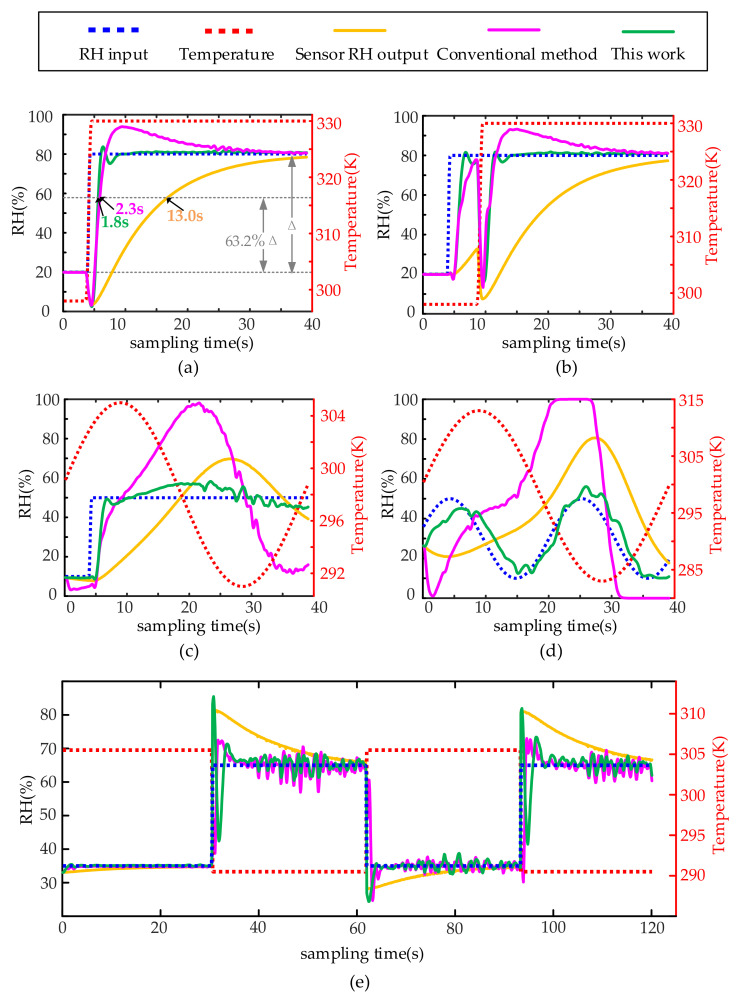
Simulation compensation results for not-constant temperature: (**a**) simultaneous step change in ambient temperature and humidity, (**b**) time-division step change in ambient temperature and humidity, (**c**) periodic change in ambient temperature and humidity step change, (**d**) the ambient temperature and humidity change periodically at the same time, (**e**) several consecutive temperatures and humidity environment switching cycles.

The second verification is carried out when temperature and humidity change at the same time. Four kinds of changes are considered, i.e., simultaneous step change of temperature and humidity, temperature and humidity staggered step change, sinusoidal temperature change and humidity step change, and temperature and humidity both sinusoidal change. The results are shown in Figure 10. Parameters of compensation filter Σ_c0_(*z*^−1^) are the same as Equation (37).

Figure 10a,b show that when the temperature and humidity change stepwise, the conventional compensation method has a significant deviation after the step change, the compensation result gradually approaches the ambient humidity after the temperature and humidity are stable for some time. Meanwhile, the proposed compensation method can yield an accurate ambient humidity value relatively quickly. Figure 10c,d show that the conventional method completely fails when the temperature has a sinusoidal change, while the proposed method can still ensure that the compensation value is close to the ambient humidity value. The response time of the sensor system in Figure 10a is 13 s, and the response time after compensation by the conventional method is 2.3 s. The response time after compensation of the proposed method is 1.8 s, which shortens the sensor response time by 86.2%. Figure 10e verifies the reproducibility of the proposed method.

### 4.2. Experiments

**Figure 11:**
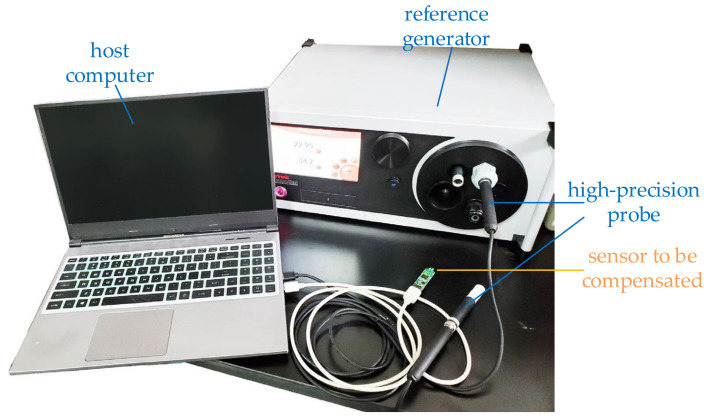
The experimental environment includes an integrated temperature and humidity sensor to be compensated, a reference generator for generating a stable temperature and humidity calibration environment, a high-precision probe for calibrating ambient temperature and humidity, and a host computer for data collection.

To verify the effectiveness of the proposed method, an experimental platform is set up, as shown in Figure 11. To generate a dynamically changing environment, the high-precision probe is switched between two steady-state temperature and humidity environments built by the air environment and the reference generator, respectively. The temperature and humidity generator used in the experiment is HG2-S of ROTRONIC Company, and the temperature and humidity sensor samples are HDC1080 of TI Company and integrated sensor samples designed by ourselves.

**Figure 12:**
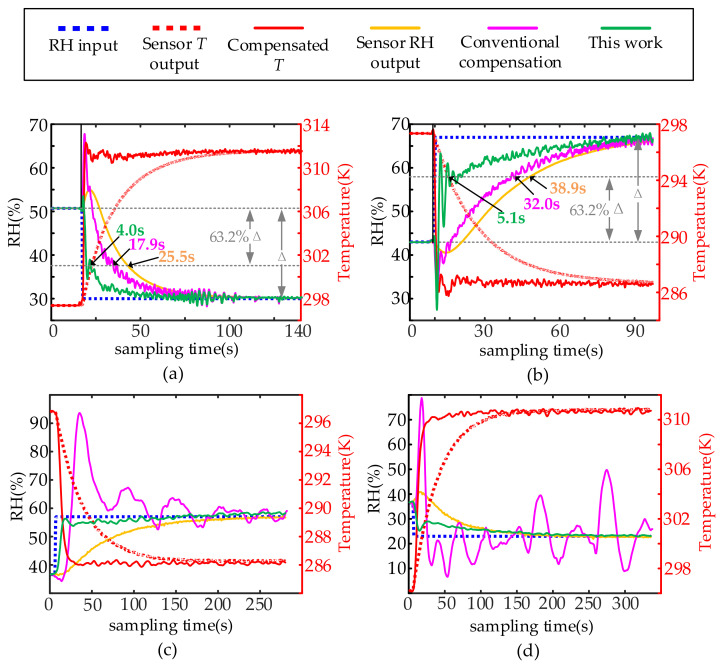
Experimental Compensation Results: (**a**) TI’s HDC1080 sensor, temperature and humidity switch from 50.7%RH (@ 297.35 K) to 30.0%RH (@ 311.65 K), (**b**) TI’s HDC1080 sensor, temperature and humidity switch from 43.0%RH (@ 297.35 K) to 67.0%RH (@ 286.25 K), (**c**) Sensors of our own design, temperature and humidity switch from 36.6%RH (@ 296.35 K) to 57.0%RH (@ 286.85 K), (**d**) Sensors of our own design, temperature and humidity switch from 36.5%RH (@ 296.25 K) to 23.0%RH (@ 311.15 K).

The results are shown in Figure 12. In the experimental verification, Figure 12a,b are the compensation results of TI’s sample HDC1080, and its compensation filter is Σ_c01_(*z*^−1^). Figure 12c,d are the compensation results of our own designed temperature and humidity sensor samples. The compensation filter is Σ_c02_(*z*^−1^). 

The parameters of the compensation filter in the experimental verification are as follows:

(38) $$\Sigma_{C_{01}}(z^{-1})=\frac{27.2436-52.3798z^{-1}+25.1512z^{-2}}{1-1.93240z^{-1}+0.94745z^{-2}}$$

(39) $$\Sigma_{C_{02}}(z^{-1})=\frac{4.02391-4.18880z^{-1}+0.42854z^{-2}}{1-1.16684z^{-1}+0.42617z^{-2}}$$

It can be seen in Figure 12 that the proposed method can perform effective dynamic compensation for the integrated temperature and humidity sensor with less distortion and faster response than that of the conventional method. The response time of the sensor system in Figure 12a is 25.5 s, and the response time after compensation by the conventional method is 17.9 s. The response time after compensation of the proposed method is 4.0 s. The corresponding response times in Figure 12b are 38.9 s, 32.0 s, and 5.1 s, respectively. From the averaged response times in Figure 12a,b, it can be seen that the proposed method reduces the response time of the humidity sensor by 85.6%, which is 19.1% using the conventional method. It can be seen from Figure 12c,d that the compensation of the traditional method has failed, and the proposed method can still perform dynamic compensation for the sensor output results.

## 5. Discussion

The dynamic compensation model proposed above is actually the condition that the recovery and response time are the same. Under this condition, the response and recovery process has the same transfer function, and the transfer function of the response process can be used to dynamically compensate the humidity sensor. However, due to the force between the moisture-sensing medium and water molecules, the response time and recovery time of some humidity sensors are not the same, especially for some fast-response humidity sensors, which use this effect to achieve rapid moisture absorption. If the proposed dynamic compensation method is to be used to compensate for this kind of humidity sensor, the process of moisture absorption and dehumidification needs to use two compensation filters with different parameters (the parameters of which need to be identified from the experimental data of the response and recovery process respectively), and it is also necessary to It is judged whether it is currently hygroscopic or dehumidified. The judgment method can select the faster one of response time and recovery time then use its corresponding compensation filter to compensate and see if the compensation result is higher or lower than the current humidity value to judge whether the compensation filter currently used is correct. If not correct, another compensation filter is used. The reason for choosing the faster one for the response and recovery time is that when we compensate for the faster process with another compensation filter (which amplifies the high-frequency noise more strongly), it creates a situation where the compensation result diverges. Unfortunately, our current research work has not been able to verify the dynamic compensation method when the response time and recovery time are different, so this part of the content is put in the discussion.

The long-term stability of the humidity sensor is a key factor affecting its measurement accuracy, but its research is mainly in steady-state measurement. Long-term drift also affects dynamic characteristics. For example, if the sensor is stored in a high-humidity environment for a long time, it will not be able to return to a dry state, and the influence of this phenomenon can be basically eliminated by heating. However, the influence of different external conditions on the long-term stability of dynamic characteristics, the extent to which this long-term drift is irreversible, and whether it affects the correctness of dynamic compensation results, etc., require further research.

## 6. Conclusions

This paper proposed a new dynamic compensation method for humidity sensors based on the decoupling of temperature and humidity by analyzing the coupling relationship between humidity and temperature in capacitive humidity sensors. The proposed method was verified by simulation and experiments. The experiment results show that the response time in the experiment is reduced by 85.6%. This method effectively reduces the deviation caused by the temperature coupling factor compared with the conventional method. The proposed method can still remain accurate when the traditional method can not compensate correctly. The proposed method can be applied to various humidity sensors whose capacitance and resistance change due to the diffusion of water molecules, or to gas sensors that detect the diffusion of gas molecules, simply by adding digital circuits to existing sensors or performing digital processing on a host computer. Therefore, the proposed method can also be easily applied to existing temperature and humidity sensors. For the most commonly used polyimide capacitive integrated humidity sensors, the sensor response time can be reduced to the level required in areas such as respiratory monitoring.

## Figures and Tables

**Table 1:** Thermal diffusivity and molecular water diffusivity of some materials in polyimide-based capacitive humidity sensors.

Materials	Thermal Diffusivity (m^2^/s)	Water Molecular Diffusivity (m^2^/s)
Polyimide	6.55 × 10^−8^	2.20 × 10^−14^
Silicon	9.07 × 10^−5^	-
Silicon oxide	3.60 × 10^−6^	-

## Data Availability

The study did not report any data.

## References

[1] Farahani H., Wagiran R., Hamidon M. (2014). Humidity Sensors Principle, Mechanism, and Fabrication Technologies: A Comprehensive Review. Sensors.

[2] Liu M.Q., Wang C., Kim N.Y. (2017). High-Sensitivity and Low-Hysteresis Porous MIM-Type Capacitive Humidity Sensor Using Functional Polymer Mixed with TiO_2_ Microparticles. Sensors.

[3] Saeidi N., Strutwolf J., Marechal A., Demosthenous A., Donaldson N. (2013). A capacitive humidity sensor suitable for cmos integration. IEEE Sens. J..

[4] Jamila B., Matthias S., Hanns-Erik E., Andreas D., Ignaz E., Christoph K., Peter M.B. (2018). Polyimide-based capacitive humidity sensor. Sensors.

[5] Dokmeci M., Najafi K. (2001). A high-sensitivity polyimide capacitive relative humidity sensor for monitoring anodically bonded hermetic micropackages. J. Microelectromech. Syst..

[6] Kalkan A.K., Li H., O’Brien C.J., Fonash S.J. (2004). A rapid-response, high-sensitivity nanophase humidity sensor for respiratory monitoring. IEEE Electron Device Lett..

[7] Qi Q., Tong Z., Wang S., Zheng X. (2009). Humidity sensing properties of KCl-doped ZnO nanofibers with super-rapid response and recovery. Sens. Actuators B Chem..

[8] Zhou C., Zhang X., Tang N., Fang Y., Zhang H., Duan X. (2020). Rapid response flexible humidity sensor for respiration monitoring using nano-confined strategy. Nanotechnology.

[9] Shen D., Xiao M., Xiao Y., Zou G., Hu L., Zhao B., Liu L., Duley W.W., Zhou Y.N. (2019). Self-Powered, Rapid-Response and Highly Flexible Humidity Sensors Based on Moisture-Dependent Voltage Generation. ACS Appl. Mater. Interfaces.

[10] Dai J., Zhao H., Lin X., Liu S., Liu Y., Liu X., Fei T., Zhang T. (2019). Ultrafast Response Polyelectrolyte Humidity Sensor for Respiration Monitoring. ACS Appl. Mater. Interfaces.

[11] Kang U., Wise K.D. (1999). A high-speed capacitive humidity sensor with on-chip thermal reset. IEEE Trans. Electron Devices.

[12] Mittal U., Islam T., Nimal A.T., Sharma M.U. (2015). A Novel Sol-Gel γ-Al_2_O_3_ Thin-Film-Based Rapid SAW Humidity Sensor. IEEE Trans. Electron Devices.

[13] Tomer V.K., Nishanthi S.T., Gahlot S., Kailasam K. (2016). Cubic mesoporous Ag@CN: A high performance humidity sensor. Nanoscale.

[14] Itoh E., Takada A. (2016). Fabrication of fast, highly sensitive all-printed capacitive humidity sensors with carbon nanotube/polyimide hybrid electrodes. Jpn. J. Appl. Phys..

[15] Subbarao N., Gedda M., Iyer P.K., Goswami D.K. (2016). Organic field-effect transistors as high performance humidity sensors with rapid response, recovery time and remarkable ambient stability. Org. Electron..

[16] Lu J., Zhang Y., Li Z., Huang J., Wang Y., Wu J., He H. (2015). Rapid response and recovery humidity sensor based on CoTiO_3_ thin film prepared by RF magnetron co-sputtering with post annealing process. Ceram. Int..

[17] Lyubchik L.M. (1994). Dynamic Sensors Distortion Compensation by Means of Input Estimation Algorithms. Intelligent Components and Instruments for Control Applications.

[18] Schoen M.P. (2007). Dynamic Compensation of Intelligent Sensors. IEEE Trans. Instrum. Meas..

[19] Jafaripanah M., Al-Hashimi B.M., White N.M. (2005). Application of Analog Adaptive Filters for Dynamic Sensor Compensation. IEEE Trans. Instrum. Meas..

[20] Yu D., Fang L., Lai P.Y., Wu A. (2008). Nonlinear Dynamic Compensation of Sensors Using Inverse-Model-Based Neural Network. IEEE Trans. Instrum. Meas..

[21] Zimmerschied R., Isermann R. (2010). Nonlinear time constant estimation and dynamic compensation of temperature sensors. Control. Eng. Pract..

[22] Yang S.L., Yang R., Zha F.Y., Liu H.D., Xu K.J. (2020). Dynamic compensation method based on system identification and error-overrun mode correction for strain force sensor. Mech. Syst. Signal Processing.

[23] Fan Y., Kong D., Lin K. (2018). Accurate measurement of high-frequency blast waves through dynamic compensation of miniature piezoelectric pressure sensors. Sens. Actuators A Phys..

[24] Xu K.J., Cheng L., Zhu Z.N. (2007). Dynamic Modeling and Compensation of Robot Six-Axis Wrist Force/Torque Sensor. IEEE Trans. Instrum. Meas..

[25] Wanga L.B., Wakayama N.I., Okada T. (2005). Numerical simulation of enhancement of mass transfer in the cathode electrode of a PEM fuel cell by magnet particles deposited in the cathode-side catalyst layer. Chem. Eng. Sci..

[26] Shibata H., Ito M., Asakursa M., Watanabe K. (1996). A Digital Hygrometer Using a Polyimi Film Relative Humiditv Sensor. IEEE Trans. Instrum. Meas..

